# US regulatory considerations for low field magnetic resonance imaging systems

**DOI:** 10.1007/s10334-023-01083-1

**Published:** 2023-05-16

**Authors:** Daniel Michael Krainak, Rongping Zeng, Ningzhi Li, Terry O’Riska Woods, Jana Gut Delfino

**Affiliations:** 1 Division of Radiological Imaging and Radiation Therapy Devices, Office of Product Evaluation and Quality, Center for Devices and Radiological Health, US Food and Drug Administration, Silver Spring, MD 20993 USA; 2 Division of Imaging Diagnostics and Software Reliability, Office of Science and Engineering Laboratories, Center for Devices and Radiological Health, US Food and Drug Administration, 10903 New Hampshire Ave, Bldg 62, Silver Spring, MD 20993 USA; 3 Standards and Conformity Assessment Program, Office of Strategic Partnership and Technology Innovation, Center for Devices and Radiological Health, US Food and Drug Administration, Silver Spring, MD 20993 USA

**Keywords:** Low field MRI, US FDA, Medical device regulation

## Abstract

Although there has been a resurgence of interest in low field magnetic resonance imaging (MRI) systems in recent years, low field MRI is not a new concept. FDA has a long history of evaluating the safety and effectiveness of MRI systems encompassing a wide range of field strengths. Many systems seeking marketing authorization today include new technological features (such as artificial intelligence), but this does not fundamentally change the regulatory paradigm for MR systems. In this review, we discuss some of the US regulatory considerations for low field magnetic resonance imaging (MRI) systems, including applicability of existing laws and regulations and how the U.S. Food and Drug Administration (FDA) evaluates low field MRI systems for market authorization. We also discuss regulatory considerations in the review of low field MRI systems incorporating novel AI technology. We foresee that MRI systems of all field strengths intended for general diagnostic use will continue to be evaluated for marketing clearance by the metric of substantial equivalence set forth in the premarket notification pathway.

## Introduction

What’s old is new again. While there has been a resurgence of interest in low field magnetic resonance imaging (MRI) in recent years, low field MRI is not at all a new concept. The first MRI systems approved for clinical use in the United States in 1983 to 1985 ranged in static magnetic field strengths from 0.15 T to 1.5 T. Today, MRI systems with a wide variety of nominal static magnetic field strengths are available for clinical use in the United States. In this brief perspective, we offer insight into some of the regulatory considerations involved when the U.S. Food and Drug Administration (FDA) evaluates low field MRI systems for market authorization.

## A brief introduction to US device regulation

Regulations, requirements, and recommendations related to the safe and effective manufacturing, installation, and operation of MRI systems span multiple state and federal agencies, professional societies, and accrediting bodies. The Center for Devices and Radiological Health (CDRH) within the FDA is responsible for ensuring the safety and effectiveness of medical devices and the safety of radiation-emitting electronic products. FDA regulates firms that manufacture, repackage, re-label, and/or import these products, including MRI systems, sold in the United States. The focus of this article is on the contribution of the US FDA to ensuring safety and effectiveness of magnetic resonance imaging systems.

FDA takes a risk-based approach to medical device regulation. Medical devices are placed in one of three device classes (Class I, Class II, and Class III) based on the level of regulatory control necessary to provide a reasonable assurance of safety and effectiveness for the device. Class I devices are considered lowest risk, and Class III devices the highest risk. All devices, regardless of class, must adhere to the general control provisions of the Food, Drug, and Cosmetic Act that relate to adulteration, misbranding, device registration as well as device listing, premarket notification, banned devices, notification of repair, replacement, or refund, records and reports, restricted devices, and good manufacturing practices [1]. When these general controls alone are insufficient to assure the safety and effectiveness of a device, special controls may be added. Special controls are device class specific and may include items such as adherence to performance standards, patient registries, premarket data requirements, and special labeling requirements. In general, Class I devices must adhere to general controls, Class II to both general and special controls, and Class III devices are subject to the requirements defined by premarket approval (PMA) [2, 3] which demands a standalone demonstration that the device provides a reasonable assurance of safety and effectiveness for its stated indications. FDA’s webpage hosts publicly searchable databases of both FDA premarket approvals (for Class III devices: https://www.accessdata.fda.gov/scripts/cdrh/cfdocs/cfpma/pma.cfm) as well as premarket notifications (for Class II devices: https://www.accessdata.fda.gov/scripts/cdrh/cfdocs/cfPMN/pmn.cfm).

Devices may be classified as Class III either because they are life-sustaining or life supporting, or because the device is novel and little information about it is available. (Unclassified devices are Class III by default.) When MRI systems were first introduced for clinical use in the United States, they were considered novel, high-risk, Class III devices subject to premarket approval. In discussing the regulatory perspectives for low-field MRI systems, it is important to note that many of these first systems had static magnetic field strengths < 1.0 T (Table 1).

**Table 1:** When first introduced to the US market, MRI systems were Class III devices subject to premarket approval (PMA)

Year initial approval	PMA number	Sponsor	Static magnetic field strength
1983	P830053	Diasonics, inc	0.35 T
1983	P830051	Technicare corp	0.15 T, 0.3 T, 0.5 T, 0.6 T, 1.5 T
1983	P830052	Philips medical systems, Inc	0.15 T, 0.26 T, 0.5 T, 1.0 T, 1.5 T
1984	P830081	Siemens corp	0.35 T, 0.5 T, 1.0 T, 1.5 T
1984	P830076	Fonar corp	0.3 T
1985	P830074	General electric co	0.5 T, 1.0 T, 1.5 T

As shown in the above table, many of these initial PMA submissions were what we now consider to be low field systems. As experience and knowledge with MRI systems increased, the devices were reclassified into Class II, where they remain today. FDA’s webpage hosts publicly searchable databases of both FDA premarket approvals (for Class III devices: https://www.accessdata.fda.gov/scripts/cdrh/cfdocs/cfpma/pma.cfm) as well as premarket notifications (for Class II devices: https://www.accessdata.fda.gov/scripts/cdrh/cfdocs/cfPMN/pmn.cfm)

As experience with and knowledge about a device is gained, the classification can be changed through reclassification processes. Reclassification may be initiated by the FDA or may be pursued in response to a petition from a manufacturer or importer. Devices are reclassified from Class III if FDA determines that based on the benefit-risk profile of the device, a reasonable assurance of safety and effectiveness can be provided by either general controls (Class I) or a combination of general controls and special controls (Class II). The regulation for magnetic resonance diagnostic devices (21 CFR 892.1000, see insert) was established when MRI systems were reclassified from Class III into Class II in 1988 [4]. Today MRI systems continue to be regulated under 21 CFR 892.1000 (product code LNH) as Class II devices requiring 510(k) notification. MRI systems with nominal static magnetic field strengths from 0.064 T [5] through 7 T [6] have gained marketing clearance under this regulation via the 510(k) premarket notification pathway.
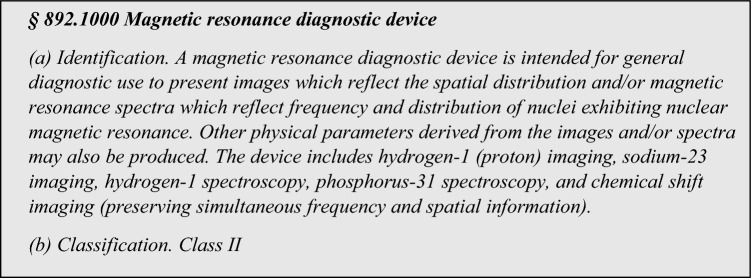


## Establishing substantial equivalence

510(k) premarket notification is the pathway to market for most Class II devices. Manufacturers must submit a 510(k) premarket notification before introducing a new device into commercial distribution for the first time or when making significant changes to a currently marketed device [7]. The 510(k) premarket notification pathway is based on a determination of substantial equivalence. All aspects of a device are considered in the determination of substantial equivalence, except for particular functions that do not meet the statutory definition of a medical device (see Multiple Function Device Products: Policy and Considerations [8]). To gain market access via the 510(k) pathway, a manufacturer must demonstrate that the new device (the subject device) is substantially equivalent to a device already on the market (the predicate device). Two devices are substantially equivalent when the new device (1) has the same intended use as the predicate device and either (2a) the new device has the same technological characteristics as the predicate device or (2b) the new device has different technological characteristics, but information demonstrates that the new device is as safe and as effective as the legally marketed device and the technological differences do not raise different questions of safety and effectiveness [9].

To demonstrate that a new MRI system is substantially equivalent to a predicate device, a manufacturer is expected to address aspects of the system’s safety—such as static field strength, acoustic output, gradient-induced electric fields, gradient-induced heating, RF energy deposition (SAR), heating of surface coils, biocompatibility of patient-contacting parts, general safety and electromagnetic compatibility—and effectiveness—such as diagnostic image quality, signal-to-noise ratio, geometric distortion, image uniformity, slice thickness, spatial resolution, and image contrast. The FDA guidance document, *Submission of Premarket Notifications for Magnetic Resonance Diagnostic Devices* [10] provides clear instructions regarding the information to be provided in a premarket notification for an MRI system. Important considerations in the determination of substantial equivalence are discussed in further detail below.

### Intended use

The intended use of a device means the general purpose of the device and its function. Two devices must have the same intended use in order to be found substantially equivalent. The indications for use (IFU) of a device is “a general description of the disease or condition the device will diagnose, treat, prevent, cure, or mitigate, including a description of the patient population for which the device is intended” [11]. Most MRI systems are brought to market with a general diagnostic intended use and all MRI systems classified under 21 CFR 892.1000 have been determined to have the same intended use. However, a future device could have an indication determined to be a new intended use that would require a different type of premarket submission for marketing authorization (e.g., a premarket approval (PMA) or De Novo Submission). In Guidance for Industry: General/Specific Intended Use [12] FDA outlines principles for when a change from a general to specific indication for use might be considered a new intended use.

### Technological characteristics

Two devices need not have the same technological characteristics to be found substantially equivalent. However, differences in the technological characteristics between the two devices cannot raise different questions of safety or effectiveness [9]. Almost all MRI systems employ a static magnetic field, a time-varying gradient magnetic field, and a radiofrequency system to generate images. All MRI systems cleared through the 510(k) pathway employ the same fundamental scientific technology (albeit with different specifications) and the differences in technological characteristics between legally marketed MRI systems classified under 21 CFR 892.1000 did not raise new questions of safety and effectiveness.

### Performance data

The performance data necessary to support a determination of substantial equivalence must demonstrate that the new device is as safe and as effective as its cited predicate device. To demonstrate safety of an MRI system, a manufacturer is expected to address questions related to static field strength, acoustic output, gradient-induced electric fields, RF energy deposition, heating of surface coils, biocompatibility of patient-contracting parts, general electrical safety, and electromagnetic compatibility. In general, all of the above are applicable regardless of the static magnetic field strength of the system. Effectiveness is assessed by the imaging capabilities of the system via phantom and clinical imaging. The user, user interface, and use environment help define use-related hazards, and appropriate human factors and useability principles apply to low-field MR systems, especially for mobile systems [13].

FDA encourages manufacturers of MRI systems to use voluntary consensus standards to support their premarket applications. Manufacturers of MRI systems may elect to declare conformity to one or more FDA-recognized consensus standards to satisfy part of a premarket review requirement [14]. For instance, most MRI system manufacturers declare conformity to *IEC 60,601–2-33, Medical electrical equipment—Part 2–33: particular requirements for the basic safety and essential performance of magnetic resonance equipment for medical diagnosis* which covers safety aspects of the MRI system. Use of other standards like the NEMA MS series can also streamline the premarket review process. While FDA strongly suggests that manufacturers use consensus standards in their premarket submissions, the use of standards is voluntary.

The current list of FDA recognized voluntary consensus standards for MRI systems (product code LNH) can be obtained from the FDA consensus standards database (https://www.accessdata.fda.gov/scripts/cdrh/cfdocs/cfStandards/search.cfm) [15].

### Benefit-risk framework

A higher static magnetic field generally increases the signal to noise ratio (and hence the image quality) of acquired images, arguably increasing the effectiveness of the system. However, a lower static magnetic field may offer potential safety advantages (for example, lower spatial field gradients that reduce the likelihood of a projectile incident, decreased RF power needs, absence of liquid cryogens and associated venting, smaller siting requirements). MRI systems have always provided a range of possibilities for clinical use, and FDA considers both benefits and risks of a device during the premarket evaluation [16].

Depending on the proposed intended use, proposed indications, and technological characteristics, it may be warranted for the indications for use for a low field MRI system to specify more particular conditions or scenarios in which the MRI system is intended to be used. Such clarification in the IFU is appropriate when the system shows a decrease in benefit (e.g., significantly lower diagnostic quality) in conjunction with some other advantage (e.g., bedside imaging) and a decrease in risk or equivalent risk. Examples of such indications for use include “where full diagnostic examination is not clinically practical” [5] or “images are not intended to be used for diagnostic purposes, and a 3 T MR image acquired without an endorectal coil is a required input” [17]. Despite the needed clarification in the IFU for such a system, the system retains the same intended use and can be found substantially equivalent under the 510(k) paradigm.

## Artificial intelligence

Artificial intelligence (AI) has quickly expanded its applications from computer vision into the field of medical imaging. A growing number of legally marketed medical devices contain AI technology. Because AI is such a new technology, no FDA guidance on its use in medical device submissions yet exists. However, FDA published a white paper in 2021 on AI/ML-based Software as a Medical Device (SaMD) Action Plan summarizing the five actions toward a practical oversight of AI/ML-based SaMD [18]. Meanwhile, FDA does make public a list of AI/ML-enabled medical devices marketed in the United States as a resource to the public [19]. The FDA list of cleared devices that include AI/ML technology is dominated by examples from the radiology panel, including many from MRI. AI can be found assisting data acquisition [20, 21], patient positioning [22], image reconstruction [23–25] and image processing (such as. de-noising) in MRI systems [26, 27].

By training a deep neural network with an ensemble of low-quality data inputs and the corresponding high-quality image targets, AI reconstruction may be used to enhance the quality of images acquired under conditions that may lead to artifact, noise, or lower signal-to-noise data, such as a lower static magnetic field. However, due to its data-driven learning mechanism, AI has generalizability concerns when processing data of a distribution different from the training data. For example, an AI reconstruction engine trained with all healthy patient data may not recover the previously unseen pathological features in a diseased patient [28, 29]. AI can also be vulnerable to adversarial attacks (known as the instability problem) due to highly nonlinear, complex, discontinuous mapping functions possibly learned as a result of training a deep neural network with limited data.

In terms of regulatory review, introduction of AI into an MRI system has been viewed as a technological characteristic that has not to date been viewed as fundamentally altering the benefit-risk profile of the system. That is, no different questions of safety and effectiveness that would preclude a comparison to the predicate device have been identified for low field MRI systems incorporating AI technology. Consider, for example, an AI-based MRI image reconstruction engine. The path to clearance is the same as that of a traditional reconstruction engine; that is, by demonstrating that images made with the AI-based reconstruction engine provide comparable image quality to a predicate device. However, the generalizability and instability issues associated with the AI-based reconstruction engine do raise the need for relevant testing to ensure safety and effectiveness, and performance of the AI-based reconstruction engine needs to demonstrate that the reconstruction engine is generalizable in different subgroups within the intended use, including addressing variations associated with different scanners, imaging protocols, anatomical regions, patient populations, and healthy and diseased patients. Robust performance is expected if AI is the only reconstruction option for the MRI system, to ensure that the new AI-based reconstruction engine does not create unexpected errors when the input data are perturbed by noise and other artifacts (such as motion, metal and shading artifacts) that may occur during data acquisition. In addition, a mitigation strategy needs to be developed and implemented in case a reconstruction failure is detected.

## A brief note about imaging agents

Gadolinium-based contrast agents (GBCAs) are often used in the context of MR imaging. FDA-approved GBCAs have no restriction or indications based on static magnetic field strength in the prescribing information. Some GBCAs mention static field strength in the notes about the mechanism of action in the clinical pharmacology section (Sect. 12) [30] or clinical study descriptions in Sect. 14 [31]. Some GBCA labels include a table of T1 relaxivities at 1.5 T (e.g., gadopiclenol), but information about relaxivities at field strengths other than 1.5 T is not available in the prescribing information. While the decision to administer GBCAs is a clinical practice decision, all MRI systems should specify if they include functionality that must be used in conjunction with a GBCA.

## Device compatibility

The safety of a patient with an implanted or accessory device cannot be assumed in any MRI system, including a low field system. At all field strengths, the MR environment presents unique safety hazards for implanted and accessory devices. Specifically, the static magnetic field of the MRI system induces displacement forces and torques on magnetic materials that may cause unwanted device movement. The pulsed radiofrequency (RF) and gradient (dB/dt) fields induce vibration and heat the device or the surrounding tissue. The magnetic and RF fields may also cause medical devices to malfunction, which can result in a failure of the device to deliver the intended therapy. Additionally, the presence of the device will induce an artifact in the MR image, and the artifact may make the exam uninformative or may lead to an inaccurate clinical diagnosis, potentially resulting in inappropriate medical action. Device safety is a complex interaction between the characteristics of the MRI system (e.g. shielding and RF frequency) and the characteristics of the implanted or accessory medical device (e.g. device length, geometry and material composition). Unique combinations create situations where any specific field interaction may be greater or lower at a lower. For example, RF induced heating is sometimes higher at 1.5 T than 3.0 T [32]. Generalizations may be misleading and it is better to consider each scanner and device combination individually to determine conditions when a patient with that device may safely be scanned.

Implanted and accessory devices come to market through their own premarket submissions, which can fall into Class I, II, or III depending on the technology and intended use of the implanted or accessory device. MRI safety concerns for an implanted or accessory device are addressed within the regulatory submission for the implanted or accessory device. All implanted medical devices, medical devices that are fastened to or carried by a patient (e.g., external insulin pump, pulse oximeter), medical devices that would reasonably be anticipated to enter the MR environment during clinical care, and all medical devices that are intended to enter the MR environment generally need to address MRI Safety. In their regulatory submission, the manufacturer of the implanted or accessory device will define the conditions for safe use as well as the MR environment in which the device can be safely used. It is the responsibility of the implanted or accessory device manufacturer to confirm that their labeling remains accurate as new systems enter the market.

The FDA guidance document *Testing and Labeling Medical Devices for Safety in the Magnetic Resonance (MR) Environment* [33] outlines the safety and compatibility assessments that should generally be conducted for implanted and accessory devices seeking MRI Safety labelling. A regulatory challenge for implanted and accessory device safety at low field is that MR Conditional device labelling has generally been field strength specific and the majority of MR Conditional labelling was developed for 1.5 T and/or 3.0 T MR systems. Whether existing test methods are translatable to a particular low field system will depend on the design of that particular system (e.g. orientation of the static magnetic field, design of the RF system). Labelling that encompasses a broad range of MRI system parameters (e.g. labelling below a certain static magnetic field strength, such as < 3.0 T) may be possible when it can be shown that risks to the patient have been adequately considered and mitigated. The challenges in developing such broad labelling are that all field interactions need to be considered and patient safety continued to be assured even as new MRI systems are introduced.

If the MR conditional labelling for a device matches the operating conditions of the MRI system in which it will be placed, a patient with that device can be scanned in that system. Scanning of patients off label (i.e., in situations where device labelling is unavailable, or device and MRI system labelling does not match) is considered a practice of medicine decision (i.e., is not regulated by FDA) and will not be addressed here.

## Investigational devices

In addition to the above requirements for the manufacturers, distributors and importers of MRI systems, FDA has an interest in ensuring that MRI systems involved in investigational studies are used in a safe manner, although the role of FDA within this sphere is different than in the premarket arena. The local Institutional Review Board (IRB) is responsible for supervising non-significant risk research studies. FDA supervises significant risk research studies, and significant risk study protocols require FDA approval via an Investigational Device Exemption (IDE) before the study can proceed [34].

The initial risk determination is made by a study investigator or the local IRB. FDA becomes involved in this process when an IRB or an investigator determines that a study is significant risk. 21 CFR 812.3(m) defines a significant risk (SR) device as one that:

1. Is intended as an implant and presents a potential for serious risk to the health, safety, or welfare of a subject;
2. Is purported or represented to be for a use in supporting or sustaining human life and presents a potential for serious risk to the health, safety, or welfare of a subject;
3. Is for a use of substantial importance in diagnosing, curing, mitigating, or treating disease, or otherwise preventing impairment of human health and presents a potential for serious risk to the health, safety, or welfare of a subject; or
4. Otherwise presents a potential for serious risk to the health, safety, or welfare of a subject.

A study involving a significant risk device is always a significant risk study, but a study involving only non-significant risk devices can also be a significant risk study [35] depending on how the devices are used. For example, non-significant risk imaging used to select patients for investigational therapy could be a significant risk study. This is why the entire study protocol needs to be evaluated when making a risk determination.

A non-significant risk study is one that does not meet the definition of significant risk.

The FDA guidance document, *Criteria for Significant Risk Investigations of Magnetic Resonance Diagnostic Devices* [36] provides additional information to study investigators using MRI systems in a research setting. The guidance document identifies the operating conditions for MRI systems that FDA considers significant risk. An MRI system that exceeds any of the following operating conditions is considered significant risk and require FDA oversight via an IDE:

- Main static magnetic field over 8 Tesla for adults, children, and infants > 1 month of age; main static magnetic field over 4 T for infants less than 1 month of age, or
- Specific absorption rate (SAR) greater than 4 W/kg whole body for 15 min, 3 W/kg averaged over the head for 10 min, or
- dB/dt sufficient to produce severe discomfort or painful stimulation, or
- Peak acoustic noise over 140 dB or
- A-weighted root mean square sound pressure level greater than 99dBA with hearing protection in place

If a study protocol does not exceed any of these operating conditions, FDA would likely consider the MRI acquisition portion of that study to have a non-significant risk and therefore under the jurisdiction of the local IRB. In general, both hardware and software development are considered to have a non-significant risk provided the operating conditions specified above are not exceeded. Additionally, investigations conducted using commercially available MRI systems used in accordance with their cleared indications for use do not require an investigational device exemption.

## Conclusions

Although there has been a resurgence of interest in low field MR systems in recent years, low field MRI is not a new concept. FDA has a long history of evaluating the safety and effectiveness of MRI systems encompassing a wide range of field strengths. Many systems seeking marketing authorization today include new technological features (such as AI), but this does not fundamentally change the regulatory paradigm for MRI systems. General purpose MRI systems of all field strengths will continue to be evaluated for marketing clearance by the metric of substantial equivalence set forth in the premarket notification pathway.

## Data Availability

The author confirms that all data generated or analysed during this study are included in this published article. Furthermore, primary and secondary sources and data supporting the findings of this study were all publicly available at the time of submission.
